# Prevalence of intracranial artery stenosis in Iranian patients with acute ischemic stroke using transcranial Doppler ultrasonography

**Published:** 2016-07-06

**Authors:** Abdolhamid Shariat, Leila Niknam, Sadegh Izadi, Alireza Salehi

**Affiliations:** 1 Shiraz Neuroscience Research Center, Clinical Neurology Research Center, Department of Neurology, Shiraz University of Medical Sciences, Shiraz, Iran; 2 Clinical Neurology Research Center, Department of Neurology, Shiraz University of Medical Sciences, Shiraz, Iran; 3 Shiraz Neuroscience Research Center, Shiraz University of Medical Sciences, Shiraz, Iran; 4 Department of Epidemiology, School of Health, Shiraz University of Medical Sciences, Shiraz, Iran

**Keywords:** Ischemic Stroke, Intracranial Arteries, Transcranial Doppler Ultrasonography, Atherosclerosis

## Abstract

**Background:** The aim of this study is to determine the frequency of intracranial artery stenosis in patients with acute ischemic stroke in Iran.

**Methods:** A total of 169 patients with acute ischemic stroke were eligible to participate and were enrolled in this study from January 2012 to February 2013. All the patients were admitted to the Nemazee ‎Hospital, affiliated to Shiraz University of Medical Sciences, Iran. They underwent transcranial Doppler (TCD) ultrasonography. Mean flow velocity (MFV) of basilar artery, vertebral artery, middle cerebral artery (MCA), anterior cerebral artery (ACA), and posterior cerebral artery (PCA) were evaluated.

**Results:** A mean of patients’ age was 67.80 ± 8.14 years. There were 83 men (49.1%) and 86 women (50.9%). Overall, 43 patients (25.4%), with a mean age of 66.7 ± 6.2 years, had intracranial stenosis. The number of men and women with intracranial stenosis was comparable (52.4% men vs. 47.6% women). Hypertension (P < 0.001), hyperlipidemia (P < 0.001), and diabetes mellitus (DM) (P < 0.001) were major risk factors for intracranial stenosis.

**Conclusion:** The prevalence of intracranial artery stenosis in patients with acute ischemic stroke is 25.4% which is comparable with previous reports from Iran and other Middle East countries.

## Introduction

A stroke, sometimes referred to as a cerebrovascular accident (CVA), is among the most common causes of mortality and morbidity in developed and developing countries. The incidence of stroke is increasing in developing countries in the Middle East.^1^^,^^2^ The age-adjusted incidence rate of ischemic stroke in Iranian population was 4.5 per 1000 residents.^2^ Currently, stroke is the third cause of mortality worldwide.^3^ Several factors are responsible for this increase, including modernization and lifestyle changes, smoking, and increased the prevalence of hypertension and diabetes mellitus (DM).^4^

The previous studies have reported that intracranial etiologies are responsible for the majority of stroke cases, i.e., disturbances in intracranial circulation cause most strokes.^4^ Approximately 10% (12.9%) of ischemic strokes occur secondary to atherosclerotic intracranial arterial stenosis.^5^ It has been demonstrated that the risk of developing ischemic stroke in those with middle cerebral arteries stenosis in about 24% in 6-year follow-up.^6^ As the investigation of intracranial artery stenosis is highly important, some studies have evaluated cerebral hemodynamics and the cerebral blood flow to predict ischemic CVAs.^7^ It has been suggested that the patients with more than 50% intracranial arteries stenosis should undergo more rigorous prevention strategies.^8^

Several methods including transcranial Doppler (TCD) ultrasonography, cerebral angiography, computed tomography angiography (CTA), and magnetic resonance angiography (MRA) have been introduced to evaluate the intracranial blood flow in asymptomatic patients.^9^ Among these methods, TCD is an available, simple and non-invasive method for the assessment of the intracranial blood flow and hemodynamic changes.^10^^,^^11^ This non-invasive technique can be used for follow-up and continuous monitoring, can be performed in a bedside setting, and also can be applied for targeted thrombolysis.^12^ The European Federation of Neurological Sciences (EFNS) summarized that TCD is very useful for assessing stroke risk of children between 2 and 16 years of age with sickle cell disease, detection and monitoring of vasospasm after subarachnoid hemorrhage, evaluation of the intracranial artery stenosis and blood flow, diagnosis of the right-to-left shunts and for monitoring the cerebral reperfusion after thrombolysis therapy of middle cerebral artery (MCA) after acute ischemic stroke.^13^ The results of TCD can assist physicians to determine preventive and therapeutic strategies.^14^

Several reports have indicated that the prevalence of intracranial artery stenosis varies between different geographical regions and is higher in blacks and Asians compared with Caucasians.^15^ Although many studies have documented that the intracranial artery stenosis can lead to ischemic stroke, some others have shown that the stenosis of extracranial arteries in thrombotic stroke is more prevalent.^16^ Caucasian patients with ischemic stroke have a higher prevalence of intracranial stenosis compared to other ethnic groups.^15^ There are few studies reported from the Middle East which have addressed intracranial arteries blood flow in ischemic stroke.^17^^,^^18^ The aim of this study is to determine the frequency of intracranial artery stenosis in patients with acute ischemic stroke in Iran.

## Materials and Methods

This prospective cross-sectional study was performed in the Neurology Section of Nemazee Hospital, affiliated to Shiraz University of Medical Sciences, Iran, from January 2012 to February 2013. The medical research ethics committee as well as Institutional Review Board of … approved the study protocol. All the patients provided their informed written consents before inclusion in the study. A total of 169 adult patients with definite diagnosis of ischemic stroke were included. The Recognition of Stroke in the Emergency Room scale (ROSIER) was used to confirm cerebral infarction, also known as ischemic stroke, as a focal neurological deficit of sudden onset that persisted beyond 24 hours in surviving patients and documented by a brain CT or an MR imaging.^19^ The patients with condition confounding the clinical presentation including previous brain injuries, the patients with tumor or other conditions mimicking stroke, and those with a cardioembolic source of stroke were excluded.

Cerebrovascular risk factors such as cigarette smoking, hypercholesterolemia [history of hypercholesterolemia and/or fasting total cholesterol level > 200 mg/dL or total triglyceride (TG) level > 200 mg/dL or low-density lipoprotein (LDL) > 130 mg/dL], hypertriglyceridemia (history of hypertriglyceridemia and/or fasting triglycerides level > 180 mg/dL), arterial hypertension [history of hypertension and/or systolic blood pressure (SBP) > 140 mmHg and/or diastolic blood pressure (DBP) > 90 mmHg, out of the acute phase, treated or not], DM [diagnosis according to the criteria of the National Diabetes Data Group (NDDG)],^20^ and associated medical diseases were assessed. The patients were also evaluated regarding the cardiovascular diseases and comorbidity, such as arrhythmias and impulse conduction disorders, mitral and/or aortic valve disease, left ventricular hypertrophy, and coronary heart disease (CHD).

We used odd/even day randomization technique to select the patients randomly. The demographic data of all the patients including age, gender, place of residence, educational status, and risk factors were recorded at the time of admission. CT scans of the brain were performed for the patients and the findings were reported based on the territory of the involved artery. 

All patients underwent TCD in the following days of admission. All TCD examinations were performed at maximum 5 days after ischemic stroke. TCD studies of intracranial arteries were performed via temporal and occipital windows using a DWL Multi-Dop T unit with a 2-MHz probe. The anterior cerebral artery (ACA), MCA, and posterior cerebral artery (PCA) were evaluated bilaterally through the temporal window. The vertebral and basilar arteries were assessed via the occipital window. The depth, peak systolic velocity (PSV), mean flow velocity (MFV), end diastolic velocity, and pulsatility index (PI) were measured for each artery separately. In this study, we considered the focal MFV (increased focal velocity in the normal range of depth for each artery) of arteries for the diagnosis of intracranial stenosis according to previously published data.^21^ Therefore, we considered MFV of ≥ 80 cm/s for MCA as abnormal in favor of stenosis. In the same way, MFV of ≥ 80 cm/s for ACA was considered as stenosis.^18^ For vertebral artery, MFV of ≥ 70 cm/s was considered as abnormal while MFV ≥ 80 cm/s was considered as stenosis for basilar.^22^ For PCA, MFV of ≥ 60 cm/s was considered as stenosis.^18^ The patients, who were not evaluated due to technical problems, were regarded as poor window and the frequency was reported. Extracranial arteries were not evaluated in this study.

SPSS software (version 16, SPSS Inc., Chicago, IL, USA) was used for data analysis. Descriptive data are presented as mean ± standard deviation (SD). Chi**-**square test was used to compare the proportional data between those with and without intracranial artery stenosis. Independent t-test was used to compare parametric data between corresponding categories. Multivariate logistic regression analyses were carried out to control the potentially confounding effect of different risk factors. A two**-**sided P < 0.050 was considered statistically significant.

## Results

A total of 169 patients with acute ischemic stroke were eligible for participation and were included to undergo TCD during a 13**-**month period. A mean of patients’ age was 67.80 ± 8.14 (ranging from 29 to 92) years. There were 83 men (49.1%) and 86 women (50.9%). Table 1 represents the demographic data of all the patients. 

**Table 1:** Baseline characteristics of the 169 patients with acute ischemic stroke

**Variable**	**Value (n = 169)**
Age (years) (mean ± SD)	67.80 ± 8.14
Sex [n (%)]	
Men	83 (49.1)
Women	86 (50.9)
Education [n (%)]	
Illiterate	145 (85.8)
Primary school	10 (5.9)
Diploma	9 (5.3)
Elementary school	4 (2.4)
Associate degree	1 (0.6)
Place of residence [n (%)]	
Village	123 (72.8)
City	24 (14.2)
Large city	22 (13.0)
Risk factors [n (%)]	
Hypertension	93 (55.0)
Hyperlipidemia	57 (33.7)
DM	54 (32.0)
Smoking	47 (27.8)

DM: Diabetes mellitus; SD: Standard deviation

The quality of the procedure was adequate in 139 patients (82.2%) but it was inadequate in 30 patients (17.8%) (poor window). Intracranial vessels could not be evaluated by TCD in 17.8% of the patients (poor window). No significant difference in the frequency of intracranial stenosis was observed between the two genders (Table 2). There is no significant difference between the right and the left MCA had stenosis in 27 patients (16.0%) and 16 patients (9.5%), respectively. 43 patients (25.4%) had stenosis in at least one of the intracranial arteries. The number of stenosis intracranial vessels was 1 in 20 patients (11.8%), 2 in 8 (4.7%), 3 in 10 (5.9%), 4 in 3 (1.8%), and 6 in 1 patient (0.6%). The anterior circulation (MCA + ACA) was involved in 64 patients (37.8%) in our study while the posterior circulation (PCA + vertebral + basilar) was involved in 17 patients (8.2%). ACA and MCA were stenosis bilaterally in 6 patients (3.5%), but only 1 patient (0.6%) had bilateral vertebral stenosis.

**Table 2:** The characteristics of patients with and without intracranial stenosis

**Variable**	**Stenosis (n = 42)**	**Normal (n = 92)**	**P**
Age (years) (mean ± SD)	66.7 ± 6.2	67.4 ± 9.1	0.635
Sex [n (%)]			
Men	22 (52.4)	50 (54.3)	0.854
Women	20 (47.6)	42 (45.7)	
Education [n (%)]			0.718
Illiterate	37 (88.1)	76 (82.6)	
Primary school	3 (7.1)	5 (5.4)	
Diploma	1 (2.4)	3 (3.3)	
Elementary school	1 (2.4)	7 (7.6)	
Associate degree	0 (0.0)	1 (1.1)	
Place of residence [n (%)]			0.700
Village	7 (16.7)	13 (14.1)	
City	5 (11.9)	16 (17.4)	
Large city	30 (71.4)	63 (68.5)	
Risk factors [n (%)]			
Hypertension	32 (76.2)	43 (46.7)	< 0.001
Hyperlipidemia	25 (59.5)	23 (25.3)	< 0.001
DM	23 (57.8)	23 (25.0)	< 0.001
Smoking	13 (31.0)	25 (27.2)	0.795
Controlled risk factors [n (%)]			
Hypertension	9 (21.4)	39 (42.3)	0.032
Hyperlipidemia	7 (16.6)	26 (28.2)	0.026
DM	4 (9.5)	18 (19.5)	0.041
Transient ischemic attack	6 (14.3)	21 (22.8)	0.354

DM: Diabetes mellitus; SD: Standard deviation

Table 3 demonstrates the frequency of intracranial stenosis using TCD ultrasonography.

The frequency of poor window was significantly higher among the women (compared with men (26.7 vs. 8.4%; P = 0.002). The patients with intracranial stenosis had significantly higher prevalence of hypertension (76.2 vs. 46.7%; P < 0.001), hyperlipidemia (P < 0.001), and DM (P < 0.001) compared with the patients who had no stenosis (Table 2).

**Table 3:** Frequency of intracranial stenosis using transcranial Doppler (TCD) ultrasonography in 169 patients with acute ischemic stroke

**Variable**	**Unilateral**	**Bilateral**
ACA [n (%)]	27 (15.9)	6 (3.5)
MCA [n (%)]	37 (21.9)	6 (3.5)
PCA [n (%)]	2 (1.2)	0 (0.0)
Vertebral [n (%)]	6 (3.5)	1 (0.6)
Basilar [n (%)]	5 (2.9)	-

ACA: Anterior cerebral artery; MCA: Middle cerebral artery; PCA: Posterior cerebral artery; TCD: Transcranial Doppler

## Discussion

This study suggests that the hypertension is the most common risk factor for cerebrovascular diseases as it was found in 55.0% of our patients following hyperlipidemia, DM and smoking. TCD findings revealed that MCA and ACA are two most common arteries which are stenosis in patients with acute ischemic stroke. 

Intracranial artery stenosis is considered among the most common causes of ischemic stroke worldwide. The incidence and prevalence of intracranial artery stenosis varies between geographical regions and ethnicities even in Asian countries.^23^ Some part of this variation may be due to different modalities and different criteria used in these studies. The pattern of its epidemiology and distribution is similar to ischemic strokes.^24^ It is more common among the Hispanics, people of African descent, and among Asians compared to Caucasians.^25^ Several studies have addressed the prevalence of intracranial artery stenosis in patients with acute ischemic stroke.^24^^,^^25^ Our findings replicate the findings of a study conducted by Zarei et al.^17^ that showed stenosis of intracranial arteries was detected in 29% of the patients with acute ischemic stroke in Iran. However, in our study, the prevalence of intracranial artery stenosis was significantly lower than the results of other previously published studies from other countries in the Middle East.^17^^,^^18^^,^^26^^-^^30^ Gujjar et al.^18^ reported a prevalence of 79% for intracranial artery stenosis in patients with ischemic stroke in Oman. 

The previous studies have reported that Asian populations have a higher prevalence of intracranial artery stenosis.^17^^,^^18^^,^^31^ Wityk et al.^15^ conducted a study in 672 patients with ischemic stroke in Pakistan and reported that the prevalence of intracranial arteries stenosis was only 12%. However, Iranmanesh et al.^16^ reported that intracranial artery diseases in Caucasians are as high as 16**-**25%. Wasay et al.^31^ reported a low prevalence of intracranial vessels in ischemic stroke in Pakistan that is similar to the low frequency of carotid artery disease in patients with stroke in Southeast Asia.^32^^,^^33^ They also reported that there is a correlation between carotid atherosclerosis and risk factors such as hypertension, smoking status, and DM that is consistent with previous studies.^32^^,^^33^ It has been reported that the prevalence of intracranial stenosis in patients with ischemic stroke is 33**-**50% in China, 47% in Thailand, 48% in Singapore, and 10**-**25% in Korea.^19^ O’Leary et al.^34^ conducted a study in the USA and showed that the prevalence of intracranial stenosis in patients with stroke and transient ischemic attack is similar to asymptomatic population.^34^ In their study, 1189 members of the Framingham cohort (asymptomatic), aged 66-93 years, were examined that showed there were no diseases in 30%, < 50% stenosis in 62%, 50-74% stenosis in 5%, 75-99% stenosis in 2%, and 100% stenosis in 1%.^34^ These results are comparable with our results in which only 25.4% of our patients with ischemic stroke had intracranial artery stenosis. In the present study, hypertension, hyperlipidemia, and DM were major risk factors for intracranial stenosis, but smoking was not. Controlling risk factors were protective against developing intracranial stenosis. The risk factors reported in this study is comparable with other previous studies.^32^^-^^34^

Zarei et al.^17^ reported that multiple intracranial stenosis were more common that single artery stenosis. Suh et al.^35^ also reported a prevalence of 21% for single stenosis, 79% for multiple stenosis, 52% for intracranial lesions, and 48% for the extracranial area in patients with ischemic stroke. Their results also showed that anterior circulation was involved in 59%, but posterior circulation was involved in 41% of the patients.^35^ In our study, the MCA was the most common site of stenosis followed by ACA. Right MCA was involved in 16% but left MCA was involved in 9.5% of our patients. In the same way, the right ACA was involved in 11.5% and the left in 7.7%. Zarei et al.^17^ showed that MCA was the most common involved artery with a prevalence of 11% bilaterally and 5% unilaterally. Gujjar et al.^18^ reported the involvement of anterior circulation in 22 patients (11.8%) and posterior circulation in 22 patients (11.8%). The anterior circulation (MCA + ACA) was involved in 64 patients (37.8%) in our study while the posterior circulation (PCA + vertebral + basilar) was involved in 17 patients (8.2%) that are higher than any other previously reported incidence rates.^18^ The prevalence of ACA and MCA stenosis was higher in our study than what has been reported by Baumgartner et al.,^36^ however, the incidence of vertebral and basilar stenosis was comparable.

It has been shown that the accuracy of TCD is the highest for the evaluation of MCA compared with other intracranial arteries which adds to the value of TCD as a non-invasive technique.^37^ When applying TCD results especially for MCA, we should keep in mind that there are other causes which can increase MFV of MCA other than atherosclerosis such as recanalization of an occluded MCA.^38^ Therefore, it is recommended that the patients with intracranial stenosis diagnosed in TCD to be followed so that they could be differentiated form recanalization of the MCA.^38^ The diagnostic accuracy of TCD should be regarded when applying it. The sensitivity and specificity for TCD have been reported 90 and 88%, respectively.^39^

Gujjar et al.^18^ reported a moderate accuracy for TCD in determining the pathology of the intracranial vessels. They reported some abnormality in about two-thirds of their study population when using TCD, while only 56% of those patients had changes in arteries corresponding to infarct location. The findings of these studies are in consistence with other studies.^36^^,^^40^ The incidence of poor window in our study was 18.3% that is comparable to previous studies from Western countries (11-20%).^36^^,^^40^^,^^41^ However, it is less than the East Asian populations (37%).^42^ Gujjar et al.^18^ reported a moderate sensitivity of TCD compared with MRA, with a relatively higher specificity. Alexandrov et al.^21^ compared the results of TCD with conventional angiography in a group of 84 patients with acute ischemic stroke. They reported high sensitivity (87.5%) and specificity (88.6%), together with high positive (87.5%) and negative (88.6%) predictive values.^21^

A study compared the MRA and transcranial color-coded Doppler sonography in 135 MCAs among 120 patients with acute stroke and reported that angle-corrected velocities correlated well with different grades of stenosis on MRA (P = 0.006). An angle corrected MCA PSV of > 120 cm/s correlated with MRA evidence of intracranial stenosis with high specificity (90.5%) and positive predictive value (93.9%), but relatively low sensitivity (66.7%; 95% confidence interval = 61.2-69.5%) and negative predictive value (55.1%).^31^

We note some limitations to our study. First, Doppler studies are operator-dependent, and the skill of the operator plays an important role in TCD results. To solve this problem, we had one single neurology resident interpret all the TCDs. Second, we did not record the outcome of the patient to correlate the TCD results with the severity and outcome of the ischemic stroke. In addition, we did not get the follow-up data of the patients and thus the predictive value of TCD could not be interpreted. Third, we performed conventional CT-scans of the brain at the time of admission and did not repeat the imaging in the following hours. This might lead to the high frequency of not formed ischemic region in brain CT-scans. Fourth, we did not perform any other vascular imaging of the brain other than TCD. Thus, the results of TCD could not be correlated with other neuroimaging techniques such as MRA or CTA. Because we used only MFV for the diagnosis of stenosis and MFV may not increase in severe stenosis so we may not detect some cases with very severe stenosis. 

## Conclusion

The prevalence of intracranial artery stenosis in patients with acute ischemic stroke diagnosed by TCD is 25.4% which is comparable with previous reports from Iran and other Middle East countries.

## Notes:

**How to cite this article:** Shariat A, Niknam L, Izadi S, Salehi A. Prevalence of intracranial artery stenosis in Iranian patients with acute ischemic stroke using transcranial Doppler ultrasonography. Iran J Neurol 2016; 15(3): 133-9.
